# Accurate and fast estimation of taxonomic profiles from metagenomic shotgun sequences

**DOI:** 10.1186/1471-2164-12-S2-S4

**Published:** 2011-07-27

**Authors:** Bo Liu, Theodore Gibbons, Mohammad Ghodsi, Todd Treangen, Mihai Pop

**Affiliations:** 1 Center for Bioinformatics and Computational Biology, University of Maryland, College Park, USA; 2 Department of Computer Science, University of Maryland, College Park, USA; 3 Biological Sciences Graduate Program, University of Maryland, College Park, USA

## Abstract

**Background:**

A major goal of metagenomics is to characterize the microbial composition of an environment. The most popular approach relies on 16S rRNA sequencing, however this approach can generate biased estimates due to differences in the copy number of the gene between even closely related organisms, and due to PCR artifacts. The taxonomic composition can also be determined from metagenomic shotgun sequencing data by matching individual reads against a database of reference sequences. One major limitation of prior computational methods used for this purpose is the use of a universal classification threshold for all genes at all taxonomic levels.

**Results:**

We propose that better classification results can be obtained by tuning the taxonomic classifier to each matching length, reference gene, and taxonomic level. We present a novel taxonomic classifier MetaPhyler (http://metaphyler.cbcb.umd.edu), which uses phylogenetic marker genes as a taxonomic reference. Results on simulated datasets demonstrate that MetaPhyler outperforms other tools commonly used in this context (CARMA, Megan and PhymmBL). We also present interesting results by analyzing a real metagenomic dataset.

**Conclusions:**

We have introduced a novel taxonomic classification method for analyzing the microbial diversity from whole-metagenome shotgun sequences. Compared with previous approaches, MetaPhyler is much more accurate in estimating the phylogenetic composition. In addition, we have shown that MetaPhyler can be used to guide the discovery of novel organisms from metagenomic samples.

## Background

Microorganisms comprise the majority of Earth’s biological diversity, and they play essential functional roles in virtually all ecosystems [1]. In particular, human-associated microbial communities play a fundamentally important role in health and disease [2]. In many environments, however, more than 99% of the microorganisms cannot be cultured by standard techniques [3]. In order to understand the genetic diversity, population structure, and ecological roles of novel organisms, metagenomic approaches analyze the microbial genomic DNA obtained directly from the environment [4]. The number and scope of metagenomic studies have increased dramatically [5] due to the rapid advance of sequencing technologies, which enable large amounts of DNA sequencing to be performed quickly and cheaply.

**Figure 1:**
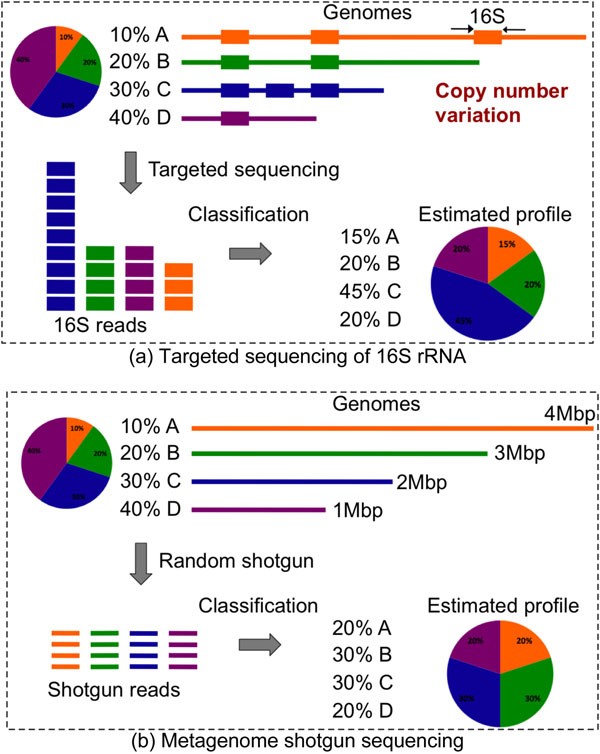
**Estimating taxonomic profiles using 16S rRNA targeted sequencing or metagenome shotgun sequencing.** Figure1a shows that the taxonomic profile estimated from 16S rRNA targeted sequencing is biased because of copy number variation. Figure 1b shows that classification of whole-metagenome shotgun sequences may produce biased estimation because of the variations in genome size.

One fundamental goal in metagenomics is to characterize the taxonomic diversity of a microbial community - taxonomic profiling. This is usually achieved by the targeted sequencing of the 16S rRNA gene, either as a whole, or focused on a hypervariable region within the gene [6]. Then the sequences are classified based on similarity against a curated reference 16S rRNA database [7]. This approach has been a powerful research tool allowing biologists to explore the majority of previously unknown microorganisms populating our world. Approaches based on 16S rRNA sequencing, however, provide a biased estimate of microbial diversity due to the wide variability in copy number of the 16S gene even within closely related organisms (Figure 1a), and due to amplification biases inherent in PCR.

A more direct approach for taxonomic profiling is to classify metagenomic reads through homology search against a reference genes database. MEGAN [8] maps query sequences to the NCBI *nr* database using BLAST, and assigns them taxonomic labels according to the lowest common ancestor of the top database hits. CARMA [9] first searches for conserved Pfam domains and protein families within the unassembled reads of a sample, then constructs a phylogenetic tree of each matching Pfam family and the corresponding query reads, and finally the reads are classified into a higher-order taxonomy depending on their phylogenetic relationships with respect to the database sequences that have known taxonomic origins. In contrast to homology-based approaches, machine learning and statistical methods [10,11] have been used to classify DNA sequences based on DNA base composition signatures (usually k-mer frequencies).

Further, a hybrid approach PhymmBL [10] has demonstrated that the combination of machine learning (Phymm) and homology information (BLAST) produces higher accuracy than either method alone. Despite the difficulties in accurately classifying whole-metagenome shotgun sequences, the estimated taxonomic profiles may be biased because of variations in genome size (Figure 1b).

In this paper, we present a novel taxonomic profiling tool (MetaPhyler) for metagenomic sequences, which relies on 31 phylogenetic marker genes [12] as a taxonomic reference. We extend the database described by Wu and Eisen [12] by including marker genes from all complete genomes, the NCBI *nr* protein database and 60 draft genomes. One major limitation of prior methods used in this context is the use of a universal classification threshold for all genes at all taxonomic levels (e.g., BLASTP E-value=0.1 used by AMPHORA [12]). However, individual bacterial genomes and proteins can have different evolutionary rates, and metagenomic reads contain gene fragments of different lengths. We propose that better classification results can be obtained by tuning the taxonomic classifier to the length of each HSP (high-scoring segment pairs in BLAST), to the reference gene, and to the taxonomic level. Our classifier, based on BLAST, uses different thresholds for each of these parameters, which are automatically learned from the structure of the reference database. A side-effect, and an important feature of our tool, is the ability to identify novel organisms or taxa. Results on simulated metagenomic datasets demonstrate that MetaPhyler outperforms previous tools used in this context (CARMA, Megan and PhymmBL). Further, MetaPhyler is much faster than previous tools for two reasons: (1) the size of the reference database is much smaller than the NCBI *nr* database; and (2) our classifier based on BLAST bit scores involves much less computation than some previous approaches which build phylogenetic trees [9,12,13]. Finally, we present several interesting results obtained by applying MetaPhyler to the gut microbiomes of obese and lean twins [14].

## Results and discussion

### Performance evaluation using simulated datasets

#### Classification performance

**Figure 2:**
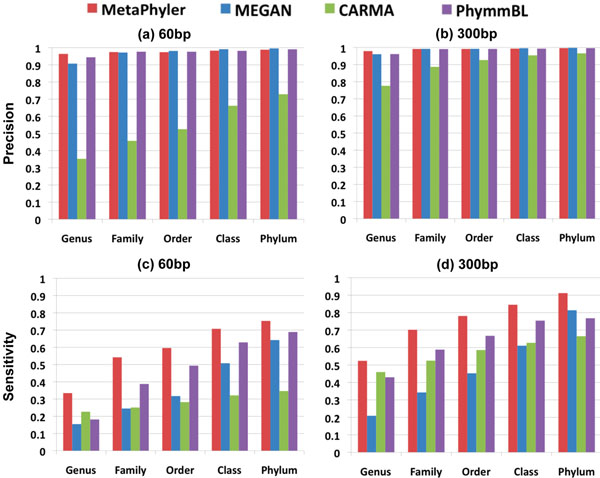
**Evaluation of classification performance** Comparison of phylogenetic classification performance of MetaPhyler, MEGAN, CARMA and PhymmBL. The sensitivity and precision are calculated across five taxonomic levels using 60bp and 300bp simulated metagenomic reads. During the classification with MetaPhyler, MEGAN, and PhymmBL, reference sequences that are from the same genome as the query reads are excluded. CARMA results are from the classifications based on WebCARMA server. This figure shows that the sensitivity of MetaPhyler significantly outperforms the other three methods, and that the precision is also slightly better at the genus level.

We carried out a simulated metagenomic study by comparing MetaPhyler with three other widely used tools: WebCarma [9], MEGAN [8] and PhymmBL [10]. We have randomly simulated around 300K 60bp and 70K 300bp DNA sequences from 31 phylogenetic marker genes. Figure 2 compares the sensitivity (*number of correct predictions* / *number of simulated reads*) *and precision* (*number of correct predictions* / *number of predictions*) of the phylogenetic assignments at five taxonomic levels. The query sequence itself was removed from the reference dataset when running MetaPhyler, MEGAN and PhymmBL. We can see that MetaPhyler, MEGAN and PhymmBL have comparable precisions in almost all cases, and MetaPhyler is a little bit better than others at the genus level. However, the sensitivity of MetaPhyler is significantly better than other tools in all situations, perhaps due to the fact that the classifiers are explicitly trained at each taxonomic level.

One of the major challenges of metagenomic analysis is the presence of novel DNA sequences which do not match well any data in current databases. One major goal of metagenomic analysis is to discover and classify such novel sequences. For example, we asked the following question: given a read from an organism whose genome has not been sequenced before, and also no sequences from the same genus are available, can we classify this sequence correctly at the family level provided that we have sequences from other organisms within the same family? We further examined the performance of MetaPhyler using progressively less data from organisms related to those from which the query sequences were simulated. Table 1 summarizes the sensitivity and precision performance evaluated on 60bp and 300bp simulated metagenomic reads. Overall the classification precision is still very high when fewer reference marker genes are available. This is especially true for the 300bp reads: even if no sequences in the database originate from the same genus as the query reads, the precision is still higher than 92% when classifying at higher taxonomic levels.

**Table 1:** MetaPhyler performance using fewer and fewer training dataset

Exclude Training	60bp					300bp				
	
	Genus	Family	Order	Class	Phylum	Genus	Family	Order	Class	Phylum
**Genome**	90.72 33.45	97.18 54.22	98.10 59.59	99.11 70.72	99.56 75.30	97.90 52.39	99.14 70.17	99.15 78.09	99.34 84.52	99.64 91.18
**Genus**		77.15 16.47	86.32 23.16	94.92 34.60	96.72 43.48		92.55 31.06	95.71 48.63	98.23 64.22	98.84 77.35
**Family**			63.62 13.19	90.31 24.64	94.65 34.99			85.25 26.65	96.78 53.15	97.66 69.42
**Order**				80.04 17.73	90.29 27.80				93.69 39.97	96.26 58.86
**Class**					78.16 16.59					90.94 42.62

MetaPhyler phylogenetic classification performance on 60bp and 300bp simulated metagenomic reads. For each prediction, the top and bottom numbers are precision and sensitivity in percentage, respectively. Different taxonomic levels are excluded when evaluating the classification, e.g., ’Genus’ means genes that have the same genus label as the query read are excluded from the reference training dataset.

#### Estimating bacterial composition

**Figure 3:**
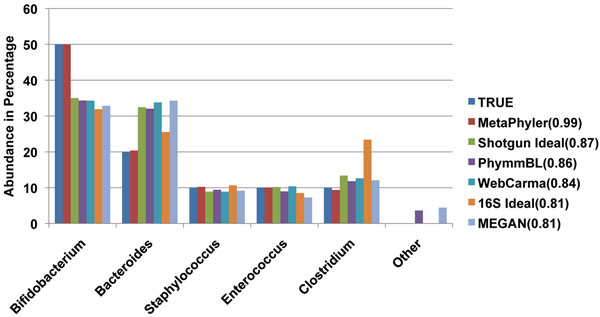
**Comparison of bacterial compositions estimated from different approaches.** We have created a simulated metagenomic sample (Table 2) with 100bp reads to evaluate the performance of different approaches in estimating the bacterial compositions. ”16S Ideal” and ”Shotgun Ideal” represent results obtained by analyzing 16S rRNA genes and whole genome shotgun sequences assuming the classification accuracy is perfect. Genus ”Other” indicates that sequences have been classified into genera other than that in the simulated sample. Different approaches are ranked by their correlation coefficients (shown in legend) between the estimated and true taxonomic profile. When running MetaPhyler, the genomes from which the reads were simulated are removed from the reference database.

As we have discussed in the Introduction section, estimating the abundance of taxonomic groups in a sample through the classification of phylogenetic marker genes is more accurate than that obtained through 16S rRNA analysis or classification of all of the metagenomic shotgun sequences. In order to validate our hypothesis, we have created a simple simulated metagenomic sample comprising 5 genomes (Table 2). We compared the accuracy of the taxonomic profiles estimated by different approaches (Figure 3). The genomes, which are present in the simulated sample, are eliminated from MetaPhyler reference database. MetaPhyler outperforms other approaches dramatically, and is very close to the true taxonomic profile. While for approaches based on classifying 16S rRNA and all the shotgun sequences, even if we assume that the classification is perfect (”16S Ideal” and ”Shotgun Ideal” in Figure 3), the resulting taxonomic profile is still highly biased.

**Table 2:** Simulated metagenomic sample

Species	Coverage	Abundance	Genome Size	# 16S rRNA
Bifidobacterium bifidum PRL2010	25	50%	2.2Mbp	3 copies
Bacteroides fragilis NCTC 9343	10	20%	5.1Mbp	6 copies
Staphylococcus aureus USA300	5	10%	2.8Mbp	5 copies
Enterococcus faecalis V583	5	10%	3.2Mbp	4 copies
Clostridium difficile 630	5	10%	4.2Mbp	11 copies

To evaluate the performance of different approaches in estimating the bacterial composition, we have created a simulated metagenomic sample consisting of 5 species with 100bp reads. ”Coverage” indicates the depth of the coverage of the simulated reads in the simulated sample for the genomes.

### Taxonomic profiling of gut microbiomes from obese and lean twins

To demonstrate the capabilities of MetaPhyler in analyzing real metagenomic datasets, we used MetaPhyler to analyze the microbial diversity of the gut microbiome from lean and obese subjects, relying on data from [14]. This metagenomic dataset comprises 6 samples from obese subjects and 6 samples from lean subjects. In the original study, the taxonomic profiles for different individuals are estimated from the analysis of full-length 16S rRNA gene, V2 and V6 hypervariable regions, and shotgun sequencing of community DNA. To compare the results with the original paper, after running MetaPhyler on the shotgun sequences, we have used the modified *t*-test [15] to compare the taxonomic profiles between obese and lean groups at the phylum level (Table 3). As a result, we have identified three phylum-level clades (out of ten) to be differentially abundant: Actinobacteria, Euryarchaeota and Verrucomicrobia. The higher proportion of Actinobacteria in obese compared with lean individuals confirm the observations in the original paper [14]. Also we have determined Firmicutes not to be differentially abundant, which agrees with the original findings. In contrast with the original study, we found the lower proportion of Bacteroidetes in obese (relative abundances are 29.80% and 21.73% in lean and obese individuals) not to be significant because of large variances within the two groups under comparison. Furthermore, we have discovered that the Verrucomicrobia bacteria are highly enriched in the lean group, which was not revealed in the original study but is consistent with the findings from another human gut metagenomic study [16]. By applying MEGAN to these data we only identified Actinobacteria (*p* = 0.02) to be enriched in obese subjects.

**Table 3:** Estimation of taxonomic profiles in obese and lean gut microbiomes

Phylum	***p *** **value**	Enriched	Notes
Actinobacteria	0.03	Obese	Confirms original study
Bacteroidetes	0.48	Lean	Large variance
Euryarchaeota	0.04	Obese	Novel
Firmicutes	0.41	Lean	Confirms original study
Verrucomicrobia	< 0.01	Lean	Confirms [16]

Comparison of taxonomic profiles, estimated by MetaPhyler, between obese and lean gut microbiomes at the phylum level.

### Detecting novel organisms

As mentioned in the Introduction (see Methods for details), MetaPhyler can help to identify novel bacteria from metagenomic sequences. Here we show a concrete example based on sample F10T1Ob1 from the above-mentioned human gut metagenome dataset. We have identified a set of reads belonging to the order Clostridiales, but novel at the family level. We then used Minimus [17] to assemble 9 reads that are mapped to the *rplB* gene. One of the resulting contigs (comprising 5 reads) contained the full-length *rplB* gene. We searched the contig against the NCBI *nr* database and identified as the best hit the *rplB* gene from species *Ruminococcussp*. *SR1*/*5* with 94% and 86% similarity at the amino acid and nucleotide levels, respectively. In addition, our assembly of another contig containing a fragment of *rplB* gene had 93% and 82% similarity with *BlautiahanseniiDSM20583* at the amino acid and nucleotide levels, respectively. Given the low level of similarity at the nucleotide level between the genes extracted from the dataset and all previously characterized genes, we can be fairly confident that the *rplB* genes we identified are novel and likely belong to previously unsequenced members of the Clostridiales order. It is important to note that this discovery was made possible by the stringent strategy we employ which avoids assigning an organism to a lower-level taxonomic group if the evidence does not support this assignment, a feature not available in other taxonomic profiling tools.

### Comparison of running times

We compared the running time of MetaPhyler with three other tools (PhymmBL, MEGAN and WebCarma) on 70K 300bp simulated phylogenetic marker gene fragments (Table 4). On a single 2.4GHz processor, the running times (including BLAST search) of MetaPhyler, PhymmBL and MEGAN for analyzing the simulated dataset are 8 hours, 4 days, and 34 days, respectively. On the same dataset WebCarma [18] took 24 hours. MetaPhyler is much faster than other tools in estimating the taxonomic compositions from metagenome shotgun sequences.

**Table 4:** Running time comparison of different tools

Dataset	CPU hours			
	
	MetaPhyler	PhymmBL	MEGAN	WebCarma
70K reads	8 hours	4 days	34 days	24 hours

On a single 2.4GHz processor, the computation time (CPU hours) used by MetaPhyler, PhymmBL and MEGAN for analyzing 70K 300bp simulated sequences. CPU hours for WebCarma are calculated using its web server.

## Conclusions

We have introduced a novel taxonomic classification method for analyzing the microbial diversity of metagenomic sequences. Compared with previous approaches, MetaPhyler provides significantly higher sensitivity when classifying 60bp and 300bp simulated reads; MetaPhyler has slightly higher classification precision at the genus level, and comparable precision at higher taxonomic levels. More importantly, the taxonomic profiles estimated by MetaPhyler are much more accurate than those estimated by other tools. In addition, MetaPhyler is much faster than other tools for taxonomic profiling because (1) the reference marker genes database is much smaller than a general reference genes database (e.g., the NCBI *nr* database), (2) and also our classifier based on BLAST statistics involves much less computation than building phylogenetic trees (another approach used for taxonomic profiling). The high performance of MetaPhyler makes it suitable for large scale metagenomic studies, e.g., the Human Microbiome Project. Furthermore, analysis of publicly available metagenomic data agrees with previous observations, and also provides new insights into the microbial diversity of the human gut ecosystem. Finally, we have demonstrated that MetaPhyler can be used to guide the discovery of novel organisms from metagenomic sequences.

The novel classification algorithm for short DNA reads we have introduced in this paper can also be applied to other conserved genes. We are planning to release a general gene fragment classifier, which can learn classification thresholds automatically from a user provided dataset. In addition, instead of providing a binary result for each classification, we will also explore techniques for generating ”fuzzy” classifications based on confidence scores. The software described in this paper is freely available under an open-source license from http://metaphyler.cbcb.umd.edu.

## Methods

### Building a reliable phylogenetic marker genes database

To use metagenomic sequences for taxonomic profiling, we analyzed 31 protein coding marker genes previously shown to provide sufficient information for phylogenetic analysis [12]. These phylogenetic marker genes are universal, present only once in most genomes, and are rarely subject to horizontal gene transfer. Hence, they provide a more accurate estimation of the microbial composition than methods relying on 16S rRNA alone. In order to create an accurate and comprehensive reference dataset, we used the manually curated marker genes from AMPHORA as a seed dataset, and extended them by including marker genes from all complete genomes, the NCBI *nr* protein database and 60 draft genomes. Specifically, we first build MetaPhyler classifiers (see below) on the seed dataset, and then use them to classify potential marker genes. In addition, we have also included phylogenetic marker genes from Archaea, whose information is not available in the seed dataset from AMPHORA. As a result, our final marker genes dataset covers 581 genera, 214 families, 99 orders, 46 classes and 27 phyla.

### Building MetaPhyler classifiers

**Figure 4:**
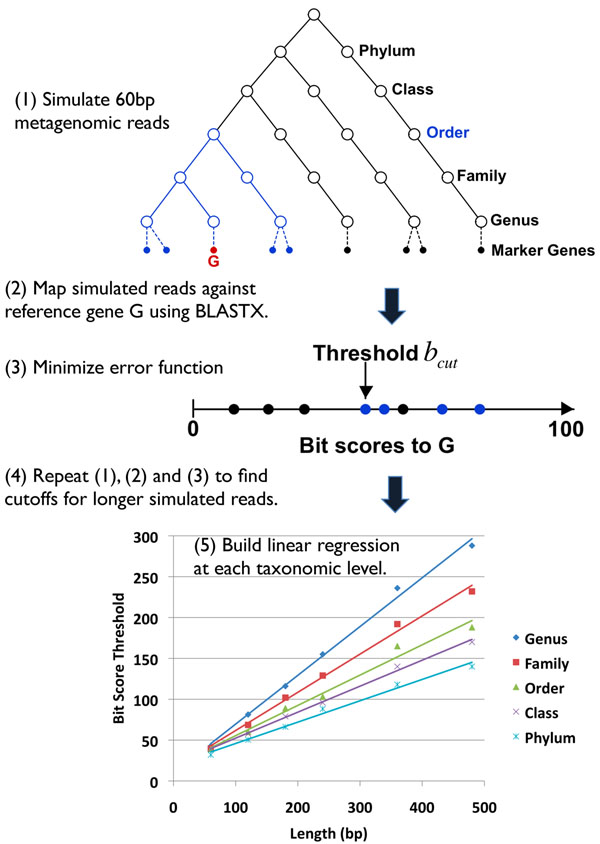
**Building MetaPhyler classifier** To build MetaPhyler for a particular phylogenetic marker gene *G* and for length 60bp, we first simulate metagenomic reads from all reference marker genes, and as a negative set, from genomic sequences that do not contain marker genes. We then map these simulated reads against reference gene *G* using BLASTX. To build a classifier for gene *G* at a specific taxonomic level, say order, in vector *B_order_* we store BLASTX bit scores between gene *G* and the simulated reads that are from the same order; in vector *B_else_* we store bit scores for aligning all other reads against G. We then find the bit score cutoff *b_cut_* that minimizes Equation 1. Finally, we repeat the previous steps to find bit score cutoffs for simulated reads of other lengths and for other genes.

Many previous metagenomic studies employ homology-based classification methods, and apply a universal threshold for all genes. The taxonomic label of the best similarity hit is then transferred to the query sequence. An improved variant of this approach involves combining the top hits instead of only using the best one [8]. We propose that better classification results can be obtained by tuning the taxonomic classifier to each BLAST HSP length, reference gene, and taxonomic rank. Specifically, by learning parameters from the reference database, we build a taxonomic classifier for a particular reference gene *G* as follows (Figure 4):

1. Simulate 60bp metagenomic reads from all reference marker genes that were curated as described in the previous section and, as a negative set, from genomic sequences that do not contain marker genes.

2. Map these simulated reads against reference gene *G* using BLASTX.

3. To build a classifier for gene *G* at a specific taxonomic level, say order, in vector *B_order_* we store BLASTX bit scores between gene *G* and the simulated reads that are from the same order; in vector *B_else_* we store bit scores for alignments of all other reads against *G*. Then, we find the bit score cutoff *b_cut_* that minimizes the following error function:(1)

where *I* is an indicator function, which equals 1 when the condition is met, and 0 otherwise. The taxonomic tree used in our analysis is downloaded from the NCBI taxonomy database, however our analysis can be redone with a different taxonomic tree.

4. Repeat the previous three steps to find bit score cutoffs for simulated reads of lengths 120bp, 180bp and up to the length of gene *G* in 60bp increments.

5. To find cutoffs for sequences of arbitrary matching lengths, we build a linear regression:  (see below for why we choose linear regression), where *L* is the sequence length,  is the bit score cutoff for length *L*, and *a* and *b* are parameters estimated from the data.

6. Repeat steps (3), (4) and (5) to build bit score cutoff regressions for other taxonomic levels (genus, family, class and phylum) for gene *G.*

We, then, repeat the above procedures to build classifiers for all reference marker genes in our database. In step (3), we assume that bit scores from close phylogenetic neighbors are higher than distant neighbors. This is generally true because marker genes, which are more closely related phylogenetically, tend to have more similar sequences. However the phylogenetic relationships of the marker genes are not fully consistent with the corresponding taxonomic tree, which is downloaded from the NCBI taxonomy database. Ideally we would expect to see the cutoff *b_cut_* to be lower than all the scores in *B_order_*, but higher than scores in *B_else_.* The error metric (Equation 1) we used is a count of the number of misclassified points, which is similar to the 2-norm distance used by SVM classifiers.

Next, we show that in step (5) linear regression is a reasonable approximation of bit scores based on the matching HSP length. As described in [19], the bit score is(2)

where *S* is the raw score of the BLAST alignment, and *λ* and *K* are parameters depending on the database. In addition, the raw score *S* equals the sum of the scores of matching amino acids [19](3)

which is the log-odds ratio of the observed and expected frequencies. For gene *G* of length *L*, we can rewrite Equation 3 as . For metagenomic read *G'* of length *L'*(*L'* ≤ *L*), which only contains a subsequence of the full-length gene *G*, the raw score . Further, if we assume that the evolutionary mutations and amino acid compositions are randomly distributed across gene *G*, then(4)

which indicates that  . Hence, we can rewrite equation 2 for a gene fragment as(5)

where  is a constant for a particular gene *G.* As a result, the bit score  of a subsequence of gene *G* is linearly correlated with the HSP length (*L'*), and we can estimate this relationship with a linear regression as in step (5).

### Classifying metagenomic sequences

The query metagenomic sequences are initially mapped to the reference marker genes using BLASTX. MetaPhyler classifies each sequence individually based on its best reference hit. For example, assume that a query sequence *Q* has gene *G* as its best hit, the BLAST bit score is *b* and the HSP length is *L.* First we try to classify *Q* at the genus level by calculating the bit score cutoff *b_cut_* of gene *G* using the pre-computed linear regression function. If the bit score is higher than the cutoff (*b* ≥ *b_cut_*), then we transfer the genus label of reference *G* to query *Q.* Otherwise, we try to classify *Q* at higher taxonomic levels (family, order, class and phylum) using level-specific classifiers built for gene *G*, until either the classification is successful at one of the taxonomic levels or the query can not be classified.

**Figure 5:**
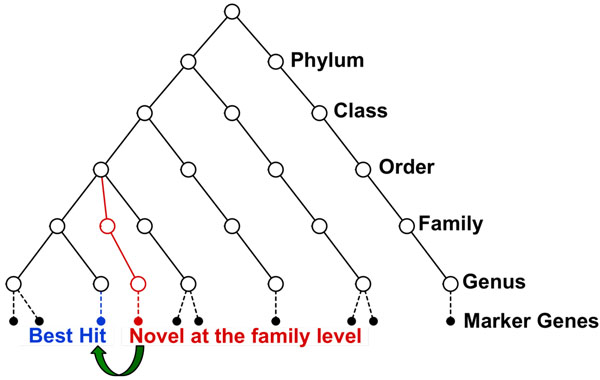
**Detecting novel organisms** Because MetaPhyler uses different classification thresholds for different phylogenetic levels, it can avoid assigning an organism to a lower-level taxonomic group if the evidence does not support this assignment. The presence of novel organisms leads to a detectable discrepancy between the number of sequences assigned to a lower taxonomic level, and the number of sequences assigned to a higher (less specific) taxonomic level.

A side-effect of this algorithm, specifically the stringent classification strategy that can avoid assigning an organism to a lower-level taxonomic group if the evidence does not support this assignment, is the ability to identify novel organisms or taxa. The presence of novel organisms leads to a detectable discrepancy between the number of sequences assigned to a lower taxonomic level, and the number of sequences assigned to a higher (less specific) taxonomic level. For example, if a set of query sequences are classified into a particular order, but cannot be classified into any existing families under this order, then this indicates that these reads come from novel family-level clades (Figure 5). These sequences can be further analyzed using a *de novo* approach, e.g., using Minimus [17], which will potentially recover the full-length gene and, thus, help characterize the novel bacterium. In order to help the users easily identity novel bacteria from MetaPhyler output, we used the following naming rule for example: if a sequence is classified at the family level as Enterobacteriaceae, but can not be classified to any genera under it, then we name this sequence as Enterobacteriaceae{family} at the genus level.

### Estimating bacterial composition

After taxonomic classification of phylogenetic marker genes from metagenomic sequences in the previous step, for each taxonomic unit, we have a set of reads assigned to each phylogenetic marker gene. The depth of coverage of this taxonomic unit is calculated as the median of that of the 31 phylogenetic marker genes. Then the relative abundances of all taxonomic units are computed using the depth of coverage instead of the number of reads classified. Table 5 shows an example of MetaPhyler output at the genus level for the simulated metagenomic sample in Table 2.

**Table 5:** An example of MetaPhyler output

Genus	Coverage	Abundance	# Reads Mapped
Bifidobacterium bifidum PRL2010	24.98	49.97%	3765
Bacteroides fragilis NCTC 9343	10.19	20.37%	1806
Staphylococcus aureus USA300	5.12	10.24%	879
Enterococcus faecalis V583	5.03	10.06%	823
Clostridium difficile 630	4.68	9.36%	748

MetaPhyler output at the genus level for the simulated metagenomic sample in Table 2.

## Authors contributions

BL and MP conceived the algorithm and designed the research and experiments. TG and MG were involved in the design of the algorithm. BL performed the experiments with the help of TT. BL and MP wrote the paper.

## Competing Interests

Authors declare that they have no competing interests.
